# Supplementary material for: BERENICE Final Analysis: Cardiac Safety Study of Neoadjuvant Pertuzumab, Trastuzumab, and Chemotherapy Followed by Adjuvant Pertuzumab and Trastuzumab in HER2-Positive Early Breast Cancer
Source: Cancers (Basel). 2022 May 24;14(11):2596. doi: 10.3390/cancers14112596 (PMC9179451; doi:10.3390/cancers14112596)
Supplement: Supplementary file 1 [file cancers-14-02596-s001.zip › cancers-1665200-supplementary.pdf]

cancers MDPI

Table S1. Timing for occurrence of first NYHA Class III/IV heart failure event.

	Adjuvant Phase	
**Total number of patients, *n* (%)**	**Cohort A: ddAC → TPH *n* = 199**	**Cohort B: FEC → DPH *n* = 198**
**≥1 heart failure event occurring after first dose of any study drug**	3 (1.5)	2 (1.0)
**Time to first cardiac event after first dose of any study drug**		
<0.5 year	3 (100.0)	0
≥0.5–<1 year	0	1 (50.0)
≥1 year	0	1 (50.0)
**Study period of first cardiac event after first dose of any study drug**		
Neoadjuvant	3 (100.0)	0
Prior to HER2 antibody therapy	0	0
During HER2 antibody therapy	3 (100.0)	0
Adjuvant	0	1 (50.0)
Treatment-free follow-up	0	1 (50.0)

For each variable, percentages are based on the number of patients in the corresponding row. Table includes cardiac events with onset from the first dose of any study drug through to the end of study, including TFFU period.
ddAC, dose-dense doxorubicin plus cyclophosphamide; DPH, docetaxel, pertuzumab, and trastuzumab; FEC, fluorouracil, epirubicin, and cyclophosphamide; HER2, human epidermal growth factor receptor 2; NYHA, New York Heart Association; TFFU, treatment-free follow-up; TPH, paclitaxel, pertuzumab, and trastuzumab.

Table S2. Timing for occurrence of first LVEF decline of ≥10% points from baseline to <50%.

	**Adjuvant Phase**	
**Total number of patients, *n* (%)**	**Cohort A: ddAC → TPH *n* = 199**	**Cohort B: FEC → DPH *n* = 198**
**≥1 LVEF decline of ≥10% points from baseline to <50% after the first dose of any study drug**	27 (13.6)	24 (12.1)
**Time to one LVEF decline of ≥10% points from baseline to <50% after first dose of any study drug**	27	24
<0.5 year	13 (48.1)	4 (16.7)
≥0.5–<1 year	10 (37.0)	12 (50.0)
≥1 year	4 (14.8)	8 (33.3)
**Study period of first LVEF decline of ≥10% points from baseline to <50% after first dose of any study drug**	27	24
Neoadjuvant	14 (51.9)	4 (16.7)
Prior to HER2 antibody therapy	0	1 (4.2)
During HER2 antibody therapy	14 (51.9)	3 (12.5)
Adjuvant	11 (40.7)	18 (75.0)
Treatment-free follow-up	2 (7.4)	2 (8.3)

For each variable, percentages are based on the number of patients in the corresponding row. Table includes LVEF significant declines with onset from the first dose of any study drug through to the end of study, including TFFU period.

ddAC, dose-dense doxorubicin plus cyclophosphamide; DPH, docetaxel, pertuzumab, and trastuzumab; FEC, fluorouracil, epirubicin, and cyclophosphamide; HER2, human epidermal growth factor receptor 2; LVEF, left ventricular ejection fraction; TFFU, treatment-free follow-up; TPH, paclitaxel, pertuzumab, and trastuzumab.

Table S3. Study treatment exposure across the neoadjuvant and adjuvant treatment periods.

	**Neoadjuvant Phase**		**Adjuvant Phase**	
	**Cohort A: ddAC → TPH *n* = 199**	**Cohort B: FEC → DPH *n* = 198**	**Cohort A: ddAC → TPH *n* = 181**	**Cohort B: FEC → DPH *n* = 190**
**Pertuzumab**	*n* = 195	*n* = 196	*n* = 180	*n* = 190
Median treatment duration, weeks (min–max)	12.0 (6.0–18.3)	12.0 (3.0–26.4)	39.0 (3.0–47.9)	39.0 (3.0–47.3)
Median number of cycles, *n* (min–max)	4.0 (2–6)	4.0 (1–6)	13.0 (1–13)	13.0 (1–14)
Number of dose interruptions/delays due to AEs, *n* (%)				
0	163 (83.6)	176 (89.8)	164 (91.1)	165 (86.8)
1	30 (15.4)	19 (9.7)	15 (8.3)	20 (10.5)
2	2 (1.0)	1 (0.5)	1 (0.6)	3 (1.6)
>2	0	0	0	2 (1.1)
**Trastuzumab**	*n* = 195	*n* = 197	*n* = 181	*n* = 190
Median treatment duration, weeks (min–max)	12.0 (6.0–18.3)	12.0 (3.0–18.0)	39.0 (3.0–47.9)	39.0 (3.0–47.3)
Median number of cycles, *n* (min–max)	4.0 (2–6)	4.0 (1–6)	13.0 (1–13)	13.0 (1–14)
Number of dose interruptions/delays due to AEs, *n* (%)				
0	154 (79.0)	170 (86.3)	165 (91.2)	164 (86.3)
1	38 (19.5)	26 (13.2)	14 (7.7)	22 (11.6)
2	3 (1.5)	1 (0.5)	2 (1.1)	2 (1.1)
>2	0	0	0	2 (1.1)

AE, adverse events; ddAC, dose-dense doxorubicin plus cyclophosphamide; DPH, docetaxel, pertuzumab, and trastuzumab; FEC, fluorouracil, epirubicin, and cyclophosphamide; TPH, paclitaxel, pertuzumab, and trastuzumab.

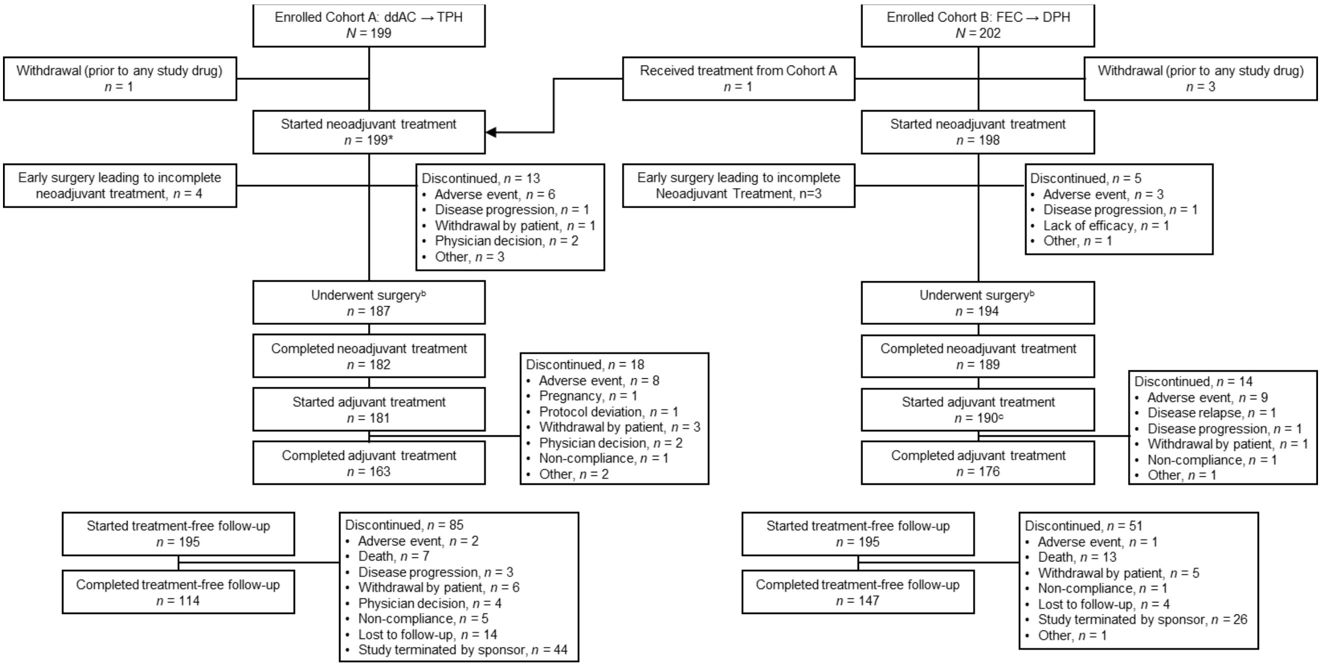

Figure S1. Patient disposition. [a] Includes one patient enrolled to Cohort B who received treatment from Cohort A; [b] Includes patients who had early surgery (incomplete neoadjuvant treatment), and patients who withdrew from the study but underwent surgery; [c] Cohort B includes a patient who did not complete neoadjuvant treatment but received adjuvant treatment; [d] The most common reason for study discontinuation was non-safety (76 patients (38.2%) in Cohort A and 37 patients (18.7%) in Cohort B), of which most were due to the end of study as per protocol (44 patients (22.1%) in Cohort A and 26 patients (13.1%) in Cohort B). ddAC, dose-dense doxorubicin plus cyclophosphamide; DPH, docetaxel, pertuzumab, and trastuzumab; FEC, fluorouracil, epirubicin, and cyclophosphamide; TPH, paclitaxel, pertuzumab, and trastuzumab.

Cohort A		Cohort B	
**Subgroup**	**5-year EFS rate (95% CI)**	**Subgroup**	**5-year EFS rate (95% CI)**
Luminal A (*n* = 33)	93.1 (83.9, 100)	Luminal A (*n* = 31)	100 (100, 100)
Luminal B (*n* = 24)	95.7 (87.3, 100)	Luminal B (*n* = 15)	93.3 (80.7, 100)
Basal-like (*n* = 11)	90.00 (71.4, 100)	Basal-like (*n* = 5)	60.0 (17.1, 100)
HER2-enriched (*n* = 80)	93.1 (87.2, 98.9)	HER2-enriched (*n* = 95)	88.3 (81.8, 94.8)

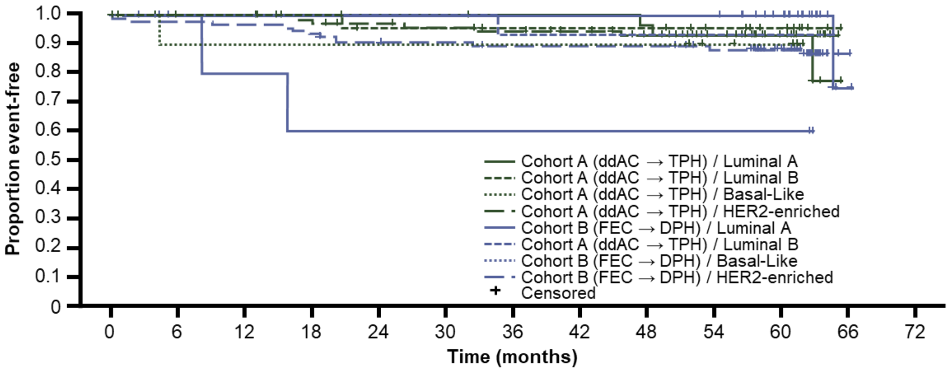

No. of patients at risk												
Cohort A (ddAC → TPH) / Luminal A	33	32	32	30	30	30	29	29	28	23	19	
Cohort A (ddAC → TPH) / Luminal B	24	23	23	23	21	21	19	19	19	16	13	
Cohort A (ddAC → TPH) / Basal-like	11	9	9	9	9	9	9	9	9	7	6	
Cohort A (ddAC → TPH) / HER2-enriched	80	78	78	73	71	69	68	65	60	46	31	
Cohort B (FEC → DPH) / Luminal A	31	28	27	27	27	27	27	27	27	27	21	1
Cohort B (FEC → DPH) / Luminal B	15	15	15	15	15	15	14	14	14	14	12	
Cohort B (FEC → DPH) / Basal-like	5	5	4	3	3	3	3	3	3	3	3	
Cohort B (FEC → DPH) / HER2-enriched	95	93	92	89	86	85	83	83	83	78	66	2

Figure S2. EFS by PAM50 classification. CI, confidence interval; ddAC, dose-dense doxorubicin plus cyclophosphamide; DPH, docetaxel, pertuzumab, and trastuzumab; EFS, event-free survival; FEC, fluorouracil, epirubicin, and cyclophosphamide; PAM50, prediction analysis of microarray 50; TPH, paclitaxel, pertuzumab, and trastuzumab.
